# Evaluating whether Prostate Cancer UK’s risk checker is a help or hindrance to prostate-specific antigen testing policy: a mixed-methods study

**DOI:** 10.3399/BJGPO.2024.0040

**Published:** 2024-06-26

**Authors:** Natalia Norori, Chiara de Biase, Yui Hang Wong, Sadie Robson Crabtree, Matt Cox, Esther Appleby, Andrew Seggie, Rachel Brown, Amy Rylance

**Affiliations:** 1 Prostate Cancer UK, London, UK; 2 Southeast London Cancer Alliance, London, UK; 3 Bristol Inner City Primary Care Network and Montpelier Health Centre, Bristol, UK

**Keywords:** prostate cancer, prostatic neoplasms, prostate-specific antigen, informed choice, male

## Abstract

**Background:**

The UK has an informed choice testing policy for prostate cancer. The prostate-specific antigen (PSA) blood test is available for free to any man aged ≥50 years who requests it and has been informed of the harms and benefits. This policy leads to differences in PSA testing rates, which can exacerbate health inequalities.

**Aim:**

To assess whether Prostate Cancer UK’s risk checker helps men at risk of prostate cancer make an informed choice about the PSA test.

**Design & setting:**

Mixed-methods study in the UK.

**Method:**

In total, 1181 men at risk, their partners, and clinical experts participated in surveys, focus groups, and one-to-one interviews. Data on risk checker completions by sociodemographic factors were analysed over time. Data from general practices that sent the risk checker to their patients were collected and analysed for service monitoring purposes.

**Results:**

There was a strong assumption that testing must be good, and therefore a need to emphasise the pros and cons of the test and that having it was the patient’s decision. Men believed their GP would invite them for PSA testing. On the impact of the risk checker, 79.6% of men who completed it had at least one prostate cancer risk factor; the average time they interacted with the information in the tool was 9 minutes 28 seconds; and 75.7% felt the tool had equipped them to make an informed choice.

**Conclusion:**

Online decision-making tools, such as the risk checker, can help reach men at high risk of prostate cancer and support them in making an informed choice about the PSA test.

## How this fits in

In the UK, the PSA test is available for free to any man aged ≥50 years but only if they request it after being informed of the potential benefits and harms. Prostate Cancer UK’s risk checker was created to support men in learning about their prostate cancer risk and making an informed choice. This study demonstrates that online decision-making tools, such as the risk checker, can effectively support men in engaging with prostate cancer information to make an informed choice about whether the PSA test is right for them.

## Introduction

Prostate cancer is the most common cancer in men. In the UK, >52 000 men are diagnosed and 12 000 die from prostate cancer every year.^1^ The UK National Screening Committee (UK NSC) recommends against organised prostate cancer screening.^2^ Instead, the official guidelines from the Prostate Cancer Risk Management Programme (PCRMP) set out an informed choice policy. It aims to support primary care professionals in providing balanced information on the harms and benefits of the prostate-specific antigen (PSA) test to asymptomatic men.^3^ The PSA test is available for free to any man aged ≥50 years who requests it and has been informed of the potential benefits and harms, but PCRMP warns that GPs should not proactively raise the issue with asymptomatic men.^3^ This creates a paradox where men can have a PSA test on the NHS, but only if they know about it and request it.

Published research suggests *‘high rates of PSA testing from “informed choice” policies in high-income countries have led to harm from overdiagnosis and overtreatment …* [While] *approaches to PSA testing that rely on people making an informed choice are likely to reflect and reproduce health inequities in preventive health care’*.^4^ In the UK, PSA testing rates vary by sociodemographic factors and are higher among men in older age groups, living in the South, and from less deprived areas on the Index of Multiple Deprivation (IMD).^5^ Furthermore, patients living in the most deprived areas are 29% more likely to be diagnosed with metastatic prostate cancer than those in the least deprived.^6^ Moreover, an analysis of stage at diagnosis data performed by Prostate Cancer UK shows that in Scotland, 35% of men diagnosed with prostate cancer are diagnosed at stage 4 compared with 21% in England.^7^

NHS data show that between April 2020 and December 2021, 14 000 fewer men received their first treatment for prostate cancer when compared with the same period in 2019, accounting for more than one-third of the reduction in cancer diagnoses during the pandemic.^8^ In response, prostate cancer was added to the Primary Care Network Contract Directed Enhanced Service (DES), with calls for an increase in the *‘proactive and opportunistic*’ assessment of patients for a potential cancer diagnosis in population cohorts where referral rates had not recovered to their pre-pandemic baseline.^9^

To address some of the issues around informed choice and pandemic recovery in prostate cancer, Prostate Cancer UK launched an online tool — the risk checker — in September 2020. The risk checker aims to support men in understanding the three main prostate cancer risk factors and to empower them to make an informed choice as to whether a PSA blood test is right for them. The tool provides evidence-based information on broad prostate cancer risk factors, highlights that most men with early stage prostate cancer are asymptomatic, and provides details about PSA blood testing, including its harms and benefits. In February 2022, Prostate Cancer UK launched an awareness-raising campaign in collaboration with NHS England signposting men to the online risk checker.

This study provides an overview of the insights that informed the content and design of the risk checker, and assesses whether it reaches men at risk and supports them to make an informed choice about the PSA test. It may also provide insights on how digital decision-making tools can address the health inequalities associated with informed choice PSA testing approaches.

## Method

**Figure 1.**
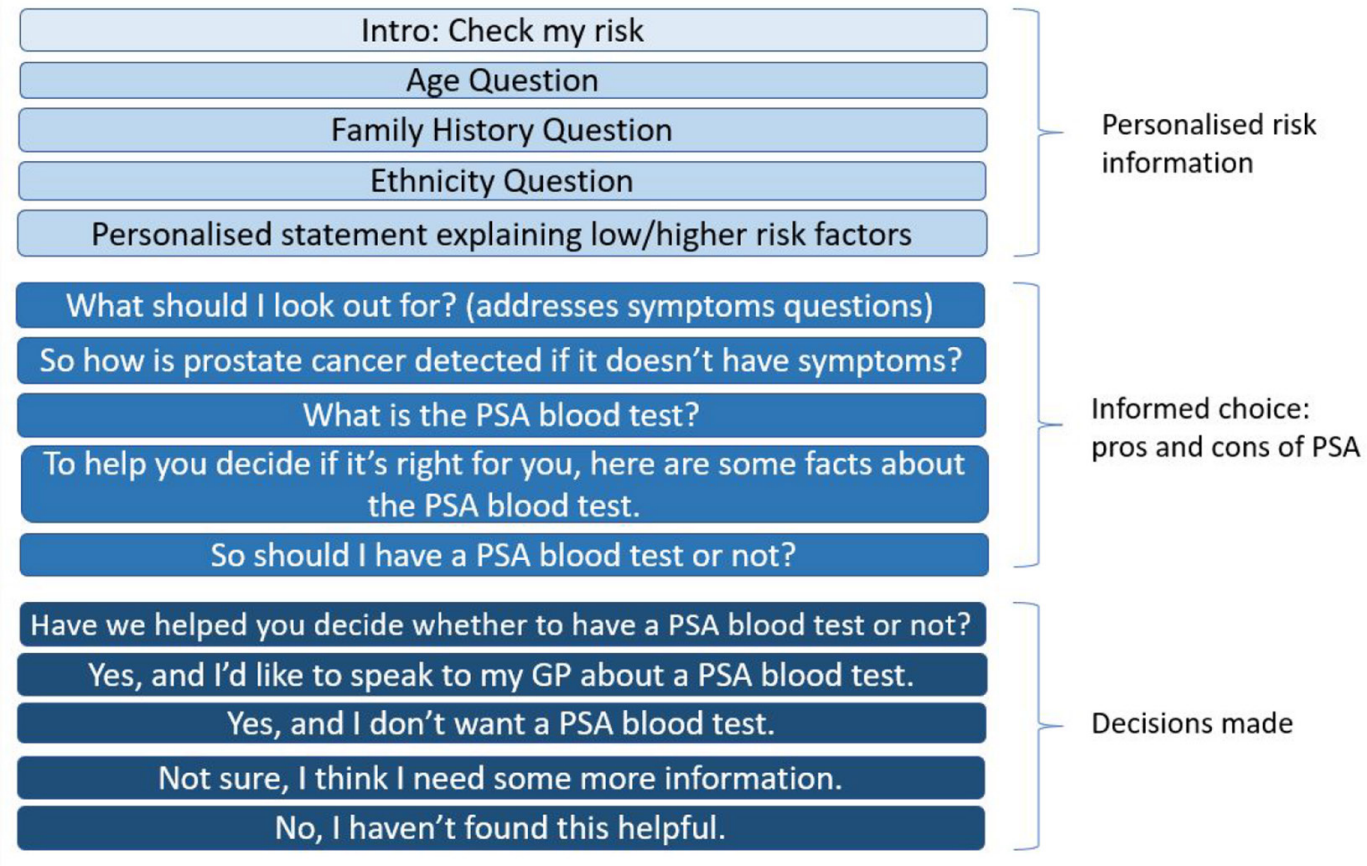
Risk checker information flow for men who have one or more of the three main risk factors for prostate cancer. PSA = prostate-specific antigen.

There have been three main iterations of the risk checker format (see Supplementary Information S1). Men with ≥1 prostate cancer risk factor are referred to as ‘men at risk’ throughout the process. The current risk checker information flow is outlined in Figure 1.

### Message testing

From March–December 2021, we engaged in message testing involving a total of 1181 participants (consisting of 1155 participants from the market research survey; 17 user-testing participants; and nine participants from the co-production workshop). This included men at risk, their partners, and clinical experts. The objective was to understand information needs, develop clearer ways of communicating risk factors for prostate cancer, and to support informed choice.

### Market research survey

In total, 1155 individuals recruited through an insights agency participated in a survey to evaluate attitudes towards prostate cancer risk and informed choice. Participants were invited via email to an online panel portal to access the survey. Consent was obtained during panel registration. The inclusion criteria required participants to be non-Black males or females aged 50–75 years, Black males aged 45–75 years, or Black females aged 45–75 years with a Black male partner. Participant characteristics are described in Supplementary Table S1. Twelve messages designed to encourage men to consider their risk of prostate cancer were assessed; eight prostate cancer awareness and risk statements and four informed choice statements (Supplementary Information S2).

### User testing

We conducted one-to-one online interviews with 17 men aged 40–71 years who did not have a diagnosis of prostate cancer. Five had a family history of prostate cancer and two were of Black ethnicity. Participants were recruited through the charity’s community awareness team and consent was sought at the start of the interview. The interviews lasted between 30 and 60 minutes, and covered the user experience and how men understood and interpreted the health messaging provided by the risk checker.

To ensure genuine reaction to the risk checker, we identified men who had little or no prior experience of prostate cancer. We recruited men in different age groups, with and without family history, and men of Black ethnicity so that all routes within the risk checker were tested. To mitigate the risk of participants being reluctant to criticise the risk checker they were told, *‘please keep in mind throughout the test that this is not a test of you — it is a test of the website. If you can’t figure something out, that’s not your fault, it’s the website’s fault — and finding those faults is exactly why we’re here. Please don’t hold anything back*.*’* The full topic guide is outlined in Supplementary Information S3.

### Co-production workshop

We brought together six clinical experts from primary and secondary care (charity contacts), and three men at risk of prostate cancer (recruited through community awareness team). The workshop discussed findings from the survey and user testing, and provided a platform for sharing knowledge and developing effective communication strategies.

### Quantitative data collection and analysis

Data on risk checker completions and trends from September 2020–May 2023 were analysed over time. This included responders’ age range, ethnicity, family history, completion rate of the risk checker tool, and time spent interacting with the tool, as well as informed choice decision. Differences in proportions were compared using χ^2^ tests. The average time interacting with the tool was calculated using Google Universal Analytics.

### Real-world case studies

General practices from Bristol Inner City Primary Care Network (PCN) and from a London integrated care system (ICS) sent text messages with a link to the risk checker to some of their higher-risk patients. Higher-risk patients were identified in line with the PCN DES definition (age, ethnicity, and family history), and ethics approval was not required because the text message constituted the contractual requirement to carry out *‘proactive and opportunistic assessment of patients’* at higher risk of prostate cancer.^9^

The Bristol general practices contacted men aged 50–70 years, Black men, and men with a recorded family history of prostate or breast cancer aged 45–70 years, in line with the PCN DES guidance, using the registered contact phone number. Men with a diagnosis of prostate cancer, those who had a PSA test within the past 12 months, and those on the end-of-life pathway were excluded. The London practices used the registered contact number to text men aged ≥45 years who were Black or had a recorded family history of prostate cancer, except for one PCN, which also contacted men aged ≥50 years.

While each general practice was free to write their own text, the suggested wording was:

Hello [Forename]. [Practice name]’s records suggest you’re at higher risk of prostate cancer. They’re inviting you to check your risk using Prostate Cancer UK’s risk checker https://prostatecanceruk.org/gp-risk-DEMO If, after checking your risk, you want a blood test then please call the practice to arrange one. [Doctor name]

In Bristol, men could call the practice and an administrator would produce the form for the patient to pick up and have the test in a phlebotomy clinic without the need for a GP appointment. In London, patients were able to reply to the text message requesting a PSA test. This enabled them to collect a PSA blood test form without needing an appointment. The text messages were coded in the GP IT system (EMIS) to enable searches to be done.

In total, 3000 patients were contacted in Bristol between July 2022 and July 2023, and 27 788 were contacted in London between December 2022 and July 2023. Data on number of PSA tests and prostate cancer diagnoses were analysed. Data were collected for service monitoring purposes.

## Results

### Message testing

The phrase *‘one in eight men will be diagnosed with prostate cancer in their lifetime’* conveyed likelihood and, as a small number, it had strong personal relevance. The *‘one in four lifetime risk for Black men’* resonated with that demographic but distanced men of other ethnicities who did not relate to that figure.

Statements about prostate cancer being *‘common’* surprised people but lacked the same personal connection as the lifetime risk statistics. Generic cancer terminology and words such as *‘die’* or *‘affects’* provoked worry without improving engagement.

The idea that a test might cause harm was deeply counterintuitive and people were surprised that they were expected to make a choice around PSA testing; for example, *‘it sounds as if having a test is not always a good idea which feels a bit odd as usually we are encouraged to take tests.’* Helpful phrases were specific about risk factors, making it memorable and personally relevant, and highlighted the ‘pros and cons’ of the PSA test, clearly signposting the need for active evaluation.

### User testing

The risk checker was perceived as a useful tool to help understand prostate cancer risk. *‘For someone like me, the average person, I would say it’s a good tool, worth going on, easy to use.’* Nonetheless, there was a need for a clearer, more decisive call to action to follow the information on risk factors. *‘Well am I at risk? Feels like I’m wading through.’* Participants highlighted the need for more detailed information on the importance of early diagnosis.

All men expressed an interest to learn more about prostate cancer after completing the risk checker. There was a strong assumption that medical tests are always beneficial, and Prostate Cancer UK’s narrative around the PSA test having benefits and harms was therefore surprising. The idea of a test having ‘disadvantages’ drove further engagement as men were curious to understand how: *’How can there be disadvantages of a test?’*

The risk checker was perceived as easy to use and helpful. *’This is really important for prostate cancer for something that affects so many men and people my age, if they want to find out if they don’t know anything about it, then it’s really useful for that.’* However, men highlighted concerns: *’I’d want information about symptoms.’ ‘What are the symptoms? Why aren’t they mentioned?’*

### Co-production workshop

Participants agreed to talk about the PSA test as the first step in the prostate cancer diagnostic pathway to enable them to decide whether they wanted a test.

Men held the expectation that their GP would recommend PSA testing once they became eligible and were surprised it was their ‘choice’. Participants agreed the need to emphasise ‘choice’ and ‘decision’ language to clarify who decides. Men expressed a strong desire to be informed about specific warning symptoms that should prompt them to speak to their GP. It was explained that most early prostate cancer does not have symptoms. However, the men participating felt strongly that we had to answer questions about symptoms up front.

After the co-production workshop, the risk checker language was considerably updated to incorporate insights. For example, user testing highlighted that the order of the information did not reflect the priority questions that men had, which made it feel like *’wading through’* to find answers to their questions. The co-production workshop agreed the sequencing of information to ensure it reflected men’s priorities. Feedback around lack of information on symptoms in user testing and co-production resulted in new content being added, which explicitly stated that most men with early prostate cancer won’t have symptoms, but provided information about possible symptoms that should prompt a man to speak to their GP. The resulting ‘version 3’ of the risk checker has been used by >1 million men.

### Impact of the risk checker

#### Risk checker characteristics

Between September 2020 and May 2023, there were 1 356 336 risk checker completions. Version 1 was completed by 157 975 (11.6%) people between September 2020 and March 2021 (data not shown). Version 2 was completed by 118 332 (8.7%) people between April 2021 and December 2021, and there were 1 080 029 (79.6%) completions between January 2022 and May 2023 for version 3 (Table 1).

**Table 1.** Risk checker user characteristics

Characteristics	Version 2		Version 3	
	April 2021–December 2021		January 2022–May 2023	
	*n*	%	*n*	%
**Total population**	118 332	100.0	1 080 029	100.0
**Men with prostate cancer risk factors**	94 814	80.1	859 842	79.6
**Men without prostate cancer risk factors**	23 518	19.9	220 187	20.4
**Age, years**				
<45	15 423	13.0	135 715	12.6
45–49	10 343	8.7	107 293	9.9
50–54	16 545	14.0	178 292	16.5
55–59	17 488	14.8	186 790	17.3
60–64	19 533	16.5	165 081	15.3
65–69	17 115	14.5	132 276	12.2
≥70	21 885	18.5	174 583	16.2
**Ethnicity**				
Black or Mixed Black	4967	4.2	49 564	4.6
Not Black or Mixed Black	113 365	95.8	1 030 466	95.4
**Family history of prostate cancer**				
Yes	16 704	14.1	146 860	13.6
No	101 628	85.9	933 170	86.4

Out of those who completed the risk checker between September 2020 and May 2023, 1 079 046 (79.6%) had at least one prostate cancer risk factor. Specifically, 185 525 (13.7%) had a first-degree relative with prostate cancer, 59 829 (4.4%) had Black or Mixed Black ethnicity, and 1 050 808 (77.5%) were aged >50 years. When looking at the age breakdown of completions by men aged ≥50, 78.7% (*n* = 748 138/950 304) were aged 50–69 years versus 21.3% (*n* = 202 166) aged ≥70 years (data not shown).

We compared the characteristics of men who completed version 2 (April 2021–December 2021) and version 3 (January 2022–May 2023) of the risk checker (Table 1). When comparing version 3 to version 2, there was a decrease in the proportion of men who reported having a family history of prostate cancer (13.6% versus 14.1%, *P*<0.0001) and an increase in that of those who identified as having Black or Mixed Black ethnicity (4.6% versus 4.2%, *P*<0.0001), but the absolute differences in proportions were small.

#### Informed choice

**Figure 2.**
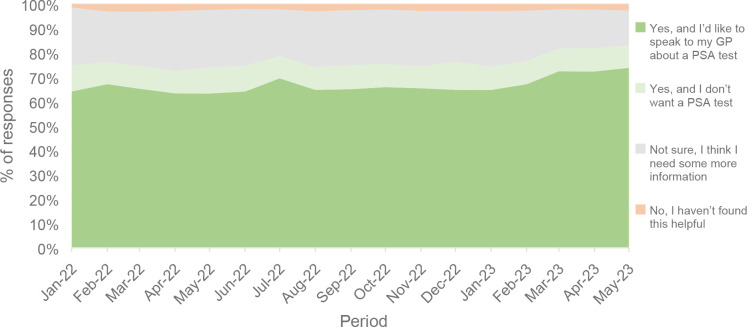
Monthly proportion of men who answered if the risk checker helped them decide whether to have a PSA blood test or not, January 2022–May 2023. PSA = prostate-specific antigen.

In total, 264 741 men provided information on the decision they had made following completing version 3 of the risk checker (30.8% of men at risk). On average, 66.3% of men reported the risk checker provided all the information they needed and decided to speak to their GP about a PSA test. Conversely, 9.4% reported the risk checker provided sufficient information and decided they did not want a PSA test. In addition, 75.7% of those completing this section felt equipped to make an informed choice, whereas 22% were uncertain (Figure 2).

#### Interaction with tool

The average time a user spent interacting with the risk checker was 9 minutes 28 seconds. This contrasts with an average engagement with health information pages on Prostate Cancer UK’s website of 4 minutes 28 seconds.

#### Real-world case studies

In Bristol, seven practices sent a total of 3000 text messages. The largest practice with a population of 20 000 patients, sent a text to 1509 of their patients. Analysis showed that 27.1% of the 1509 men contacted by that practice (*n* = 409) decided to get a PSA test, resulting in 12 new prostate cancer diagnoses (data not shown).

In London, 15 general practices sent 27 788 text messages. Of men contacted, 6233 opted for PSA testing and 365 were referred with suspected urological cancer. Of those referred with raised PSA levels, 35 (9.6%) were diagnosed with prostate cancer (data not shown). Stage at diagnosis data are currently unavailable.

## Discussion

### Summary

The findings of our message testing informed the development of the risk checker. The current version (version 3) has been completed by >1 million people. To the best of our knowledge, this study is the first to demonstrate the effectiveness of online decision-making tools in facilitating men’s understanding of prostate cancer and enabling them to make an informed choice about the PSA test.

The risk checker was created to support men in making an informed choice. Our results confirm 75.7% of men at risk who take the risk checker believe it helped them make an informed choice, whereas 21.2% were uncertain. Importantly, men are taking their time to review the information: 9 minutes and 28 seconds on average. This finding suggests the risk checker effectively meets men’s information needs and puts into perspective why providing prostate cancer information is a challenge for primary care.

Clinicians highlighted the importance of positioning the PSA test as an initial step in the diagnostic pathway, rather than as a standalone diagnostic test. The messaging now highlights the pros and cons of the entire diagnostic pathway so that men can make an informed choice of whether to get tested.

Data from the general practices have shown the risk checker can help primary care reach higher-risk patients. By responding to texts, men could book tests without an appointment, reducing additional primary care workload. Anecdotal concerns have been raised about the potential for unmanageable increases in workload for primary care services if large numbers of men aged >50 years request PSA testing, as they are a significant proportion of the UK population. Practices reported minimal workload increase and patient perceptions were positive. The risk checker helped diagnose men who may have otherwise faced delays, although further analysis on staging is needed.

The risk checker launched at a critical moment to promote informed decision making about PSA testing among men who may have experienced barriers to a PSA test during the COVID-19 pandemic. While directly measuring the impact of the risk checker on prostate cancer diagnosis and treatment was outside the scope of this analysis, real-world data have shown more men used the risk checker during the same period when more cases of urological cancer were diagnosed and treated. Analysis of NHS data have shown 67 142 patients received treatment for urological cancer from March 2022–February 2023, an 8% increase compared with pre-pandemic levels. More than half a million men completed the risk checker during this period.

### Strengths and limitations

A key strength of the study is the very large volume of data, including information from >1 million men who completed the risk checker and >250 000 who shared their resulting choice. Moreover, insights provided by 1181 participants were instrumental in supporting the risk checker development. These inputs provide valuable insights into the views of men around prostate cancer risk messaging. However, we are unable to confirm that all reported risk checker completions and informed-choice selections represent unique and genuine patients. It is possible some men may have completed the risk checker multiple times, thereby inflating the totals.

Data on the time spent interacting with the tool was calculated using Google Universal Analytics, which has been discontinued since July 2023. A limitation is that the average time on page is an aggregated figure, taken from all recorded views of the page(s) in question, and is calculated as ‘Total time on page’/(’Total pageviews’ – ‘Total exits’). Average time on page could therefore be skewed by a higher-than-average exit rate. Opportunities to click through to other pages on the Prostate Cancer UK website only appear relatively late in the risk check process, meaning recorded times could have come from users with higher levels of engagement.

### Comparison with existing literature

Our research showed men are more likely to engage with health information that feels relevant to them and their communities. While the UK currently recommends a reactive policy to prostate cancer screening, our findings highlight important issues with how this is understood by men. Unlike directive public health messaging, informed choice promotes individual decision making after weighing the benefits and harms of a diagnostic test. Interestingly, our study showed men did not associate harm with PSA testing, reflecting an assumption that medical tests are inherently beneficial. This is consistent with findings from a previous study that found men’s attitudes regarding the PSA test in the UK are overall positive.^10^

The high proportion of engagements with the risk checker being men at risk suggests targeted health messaging is weighting exposure towards those who are more likely to benefit. Research has shown older men without symptoms are unlikely to benefit from prostate cancer screening.^11^ However, in the UK, men aged 80–89 years are twice as likely to get a PSA test as men in their 50s.^4^ Considering this evidence, messaging was added to the risk checker that explained men aged >70 years are unlikely to benefit from further investigation, unless they have symptoms. Currently, 21.3% of risk checker completions are by men aged ≥70 years.

### Implications for research and practice

Prostate cancer remains a complex health issue, with new evidence continuously reshaping our understanding of screening and treatment options. Our findings demonstrate online tools, such as the risk checker, can effectively support men in engaging with prostate cancer information in a way that empowers them to make an informed choice about the PSA test. It helps the UK PSA testing informed choice policy because it takes a difficult, time-consuming task (explaining the pros and cons of the PSA test) from primary care and delivers it at men’s convenience. Our research suggests the risk checker helps reduce the inherent inequalities associated with informed choice policies. Further work should also examine the impact of the risk checker on prostate cancer outcomes and primary care workload, and validate it as a decision-making tool in controlled settings. Additionally, the messaging should continue to be evaluated to ensure it is presented in a balanced way that helps men make a fully informed choice.

## Data

The dataset relied on in this article is available from the corresponding author on reasonable request.
